# The Role of Insulin Resistance in Mediating the Association Between Proinflammatory Diets and Metabolic Dysfunction‐Associated Steatotic Liver Disease

**DOI:** 10.1002/fsn3.72203

**Published:** 2026-08-12

**Authors:** Zongbiao Tan, Haodong He, Yang Meng, Yu pu, Yanrui Wu, Jixiang Zhang, Weiguo Dong

**Affiliations:** ^1^ Department of Gastroenterology Renmin Hospital of Wuhan University Wuhan China; ^2^ State Key Laboratory of Ophthalmology, Zhongshan Ophthalmic Center Sun Yat‐Sen University, Guangdong Provincial Key Laboratory of Ophthalmology and Visual Science Guangzhou China; ^3^ Department of General Practice Renmin Hospital of Wuhan University Wuhan China

**Keywords:** Food Inflammatory Index, MASLD, NHANES, proinflammatory dietary patterns

## Abstract

The association between proinflammatory dietary patterns and metabolic dysfunction‐associated steatotic liver disease (MASLD) has been increasingly recognized; however, the underlying biological mechanisms remain unclear. This study aimed to investigate the relationship between dietary inflammatory potential and MASLD risk and to evaluate the mediating role of insulin resistance (IR)‐related biomarkers in this association. A total of 13,675 participants from the National Health and Nutrition Examination Survey were included. Dietary inflammatory potential was assessed using the Dietary Inflammatory Index (DII) and Food Inflammatory Index (FII). Insulin resistance was evaluated using the triglyceride‐glucose (TyG) index and its anthropometric‐adjusted derivatives, including TyG‐BMI, TyG‐WC, TyG‐WHtR, and TyG‐ABSI. Multivariable logistic regression models were applied to examine the associations between DII, FII, and MASLD risk, and mediation analyses were conducted to quantify the proportion of the associations explained by IR‐related biomarkers. Participants with MASLD exhibited significantly higher DII and FII scores than those without MASLD (*p* < 0.001). After adjustment for potential confounders, both DII (OR = 1.07, 95% CI: 1.04–1.10) and FII (OR = 1.04, 95% CI: 1.02–1.06). were independently associated with increased MASLD risk. Furthermore, both dietary inflammatory indices were positively associated with all evaluated IR‐related biomarkers. Mediation analyses demonstrated that IR significantly mediated the association between proinflammatory dietary patterns and MASLD, with TyG‐WHtR showing the strongest mediating effect, accounting for 73.9% of the association between DII and MASLD and 61.5% of the association between FII and MASLD. These findings indicate that a proinflammatory dietary pattern is associated with an increased risk of MASLD and that insulin resistance may represent a key pathway linking dietary inflammatory exposure to MASLD development. The results highlight the potential importance of targeting diet‐induced insulin resistance for MASLD prevention and management.

## Graphical abstract

Study overview illustrating the association between proinflammatory dietary patterns and metabolic dysfunction‐associated steatotic liver disease (MASLD), with insulin resistance biomarkers as potential mediators. Data from the National Health and Nutrition Examination Survey (NHANES 2001–2018) were analyzed, including 13,675 participants after exclusion of individuals with missing information. Dietary inflammatory potential was assessed using the Dietary Inflammatory Index (DII) and Food Inflammatory Index (FII). Proinflammatory dietary patterns were associated with increased MASLD risk through both direct effects and indirect pathways mediated by insulin resistance‐related biomarkers, including BMI, TyG, TyG‐BMI, TyG‐WC, TyG‐WHtR, and TyG‐ABSI.

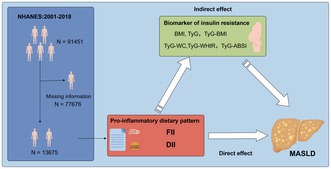

**Abbreviations**
BMI: body mass index
CI: confidence interval
DII: Dietary Inflammatory Index
FII: Food Inflammatory Index
MASLD: metabolic dysfunction‐associated steatotic liver disease
NHANES: national health and nutrition examination survey
OR: odd ratio
PIR: poverty income ratio
SII: systemic immune‐inflammation index
TyG: triglyceride‐glucose index
TyG‐ABSI: triglyceride‐glucose‐A body shape index
TyG‐BMI: triglyceride‐glucose‐body mass index
TyG‐WC: triglyceride‐glucose‐waist circumference index
TyG‐WHtR: triglyceride‐glucose‐waist‐to‐height ratio

## 1 Introduction

Metabolic dysfunction‐associated fatty liver disease (MASLD) has emerged as one of the most frequent chronic liver disorders, driven by the global epidemics of obesity, diabetes, and metabolic syndrome (Loomba and Wong 2024; Kanwal et al. 2024). Current estimates suggest that MASLD affects more than one‐third of the adult population globally, with higher prevalence in Western countries, but rapidly increasing rates in Asia, underscoring a critical public health challenge (Younossi et al. 2025).

Diet is one of the important environmental factors for the occurrence and development of MASLD (Zeng et al. 2024; Di Giosia et al. 2022; Effinger et al. 2023). Proinflammatory dietary patterns represented by high‐fat diets can induce and continuously promote inflammatory responses and oxidative stress in the body, further exacerbating insulin resistance and lipid metabolism disorders, thereby promoting the onset and progression of MASLD. Recently, two tools for comprehensive assessing dietary inflammation have been developed, including Dietary Inflammatory Index (DII) and Food Inflammation Index (FII). The DII systematically evaluate the proinflammatory properties of dietary patterns by integrating food‐derived biomarkers and nutrient profiles linked to inflammatory pathways (Shivappa et al. 2014). A prospective analysis of UK Biobank found that individuals in the highest DII tertile had a 19% higher risk of severe NAFLD (defined as hospitalization or death) compared to those in the lowest tertile (HR = 1.19, 95% CI: 1.03–1.38). (Petermann‐Rocha et al. 2023) This relationship is consistent with the evidence of many other studies (Zhao et al. 2024). The FII was recently introduced as an evaluation tool that quantifies the inflammatory potential of individual foods by integrating detailed food composition data (Wang et al. 2024). Insulin resistance is the core pathophysiological mechanism for the occurrence and development of MASLD. Currently, a series of insulin resistance biomarkers have been developed, such as triglyceride‐glucose (TyG)‐related indicators (i.e., body mass index [BMI], waist circumference [WC], waist‐to‐height ratio [WHtR], and body mass index [ABSI]). These indicators are widely used to evaluate the risk and prognosis of death from metabolic disorders and cardiovascular diseases (Tian, Chen, et al. 2022; Zhou et al. 2024). In patients with MASLD, higher TyG‐related indices are also significantly associated with all‐cause, cardiovascular and diabetic mortality, and show better predictive value in the population without advanced fibrosis. (Chen et al. 2024). At present, the association between the FII and the risk of MASLD has not been clarified, and the mediating role of insulin resistance in the underlying mechanism also awaits systematic evaluation.

Our research aims to explore the relationship between two proinflammatory dietary patterns (including FII and DII) and the risk of MASLD. In addition, we also evaluated the potential mediating role of insulin resistance biomarkers, including BMI, TyG, TyG‐WC, TyG‐BMI, TyG‐ABSI, and TyG‐WHtR.

## 2 Methods

### 2.1 Study Population

**FIGURE 1:**
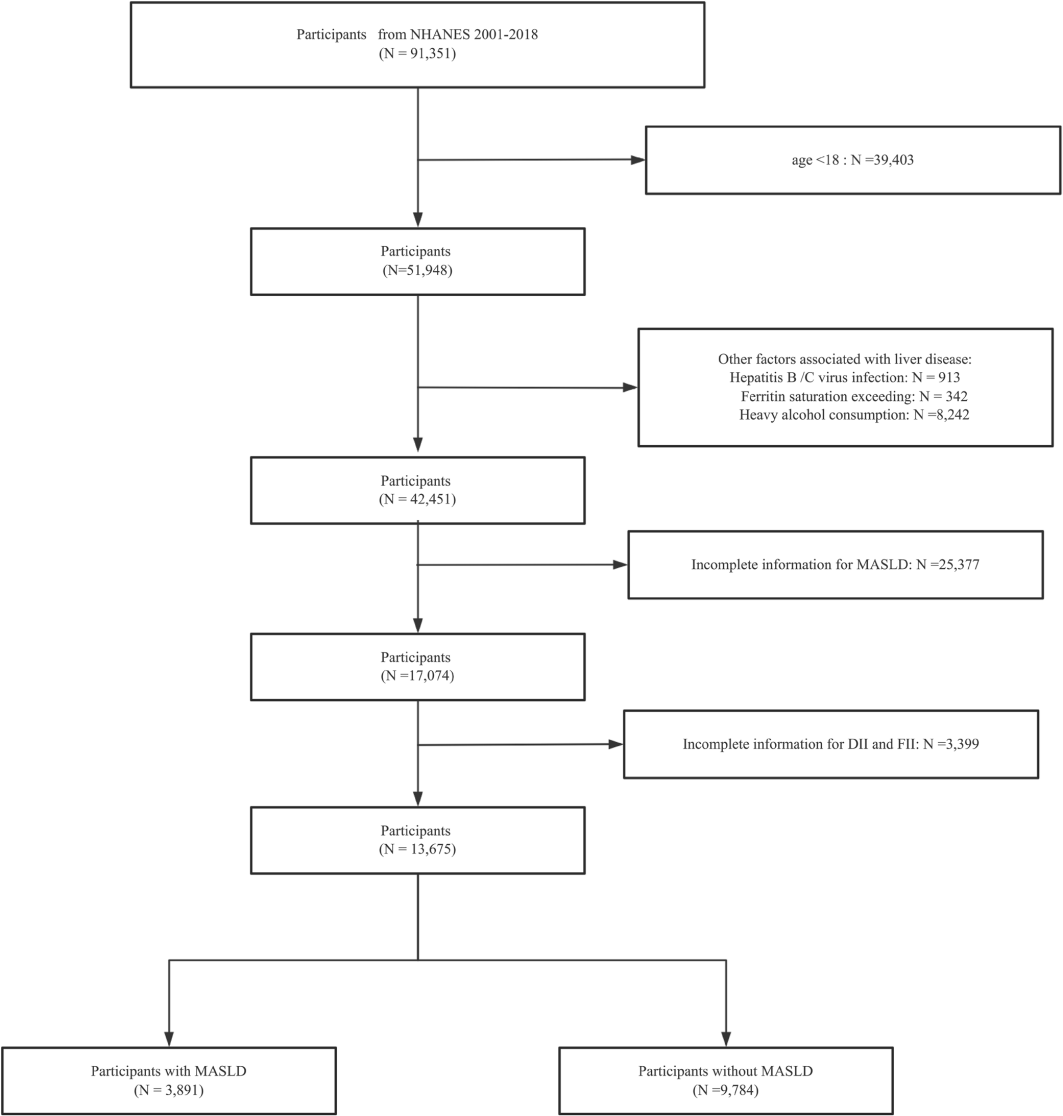
Flowchart of participant selection in the present study.

This study analyzed data from the National Health and Nutrition Examination Survey (NHANES) conducted between 2001 and 2018. Participants with missing key variables were excluded, including those with incomplete demographic information, unavailable MASLD diagnostic data, missing covariates required for multivariable adjustment, or lacking insulin resistance–related indices. The overall workflow is presented in Figure 1.

### 2.2 Definition of Proinflammatory Dietary Patterns

In this study, the DII and FII were employed as indicators of dietary inflammatory potential. DII was calculated according to the methodology established by (Shivappa et al. 2014), which systematically evaluates the overall inflammatory potential of diets by quantifying the distribution patterns of 28 dietary components with inflammatory modulation properties (including macronutrients, vitamins, and bioactive compounds). FII was derived using the method developed by (Wang et al. 2024), which calculates and compares FII scores across food groups based on the USDA Food Composition Database and dietary guidelines, thereby revealing intragroup heterogeneity in food inflammation effects. All dietary information was obtained through the Mobile Examination Center platform. A uniformly trained nutritional epidemiology research team conducted standardized data collection procedures using validated 24‐h dietary recall interviews.

### 2.3 Definition of MASLD

MASLD is defined by the presence of hepatic steatosis (≥ 5% hepatocyte fat accumulation) in conjunction with at least one of five cardiometabolic risk factors, as per the 2023 multisociety nomenclature update. Among them, hepatic steatosis is defined by a US Fatty Liver Index (US‐FLI) score ≥ 30 (Ruhl and Everhart 2015). In addition, participants with conditions that could confound liver status were excluded to improve diagnostic specificity. These included individuals with viral hepatitis, iron overload, or heavy alcohol consumption.

### 2.4 Insulin Resistance Biomarkers

Insulin resistance biomarkers included BMI, TyG, TyG‐WC, TyG‐BMI, TyG‐WHtR, and TyG‐ABSI. Detailed calculation methods are provided in Table S1.

### 2.5 Assessment of Covariates

This study incorporated demographic, lifestyle factors, and other disease variables as covariates, categorized as follows: (1). Sociodemographic variables: age, sex, race, education level, and household income (defined as a poverty‐income ratio [PIR]), (2). Lifestyle factors: smoking status, physical activity (Tian, Xue, et al. 2022), (3). Anthropometric parameters: serum alanine aminotransferase (ALT) and aspartate aminotransferase (AST). (4). Disease status: diabetes (yes/no), and hypertension (yes/no). (5). Systemic immune inflammation index (SII). For missing covariates, continuous variables were imputed using predictive mean matching (PMM), while binary variables were imputed using logistic regression models.

### 2.6 Statistical Analysis

In accordance with NHANES analytical guidelines, this study takes into account the complex multistage sampling design and incorporates appropriate sample weights to ensure nationally representative estimates. First, a descriptive statistic was stratified by participants' disease status. For continuous variables, results were reported as weighted mean ± standard error (SE) and analyzed using Student's *t*‐tests. For categorical variables expressed as weighted percentages (%) and assessed via chi‐square tests. To evaluate the relationship between FII, DII, and MASLD risk, two weighted multivariate logistic regression models were developed: partially adjusted model: adjustment for demographic covariates (age, sex, race, PIR); fully adjusted model: based on partially adjusted model adjustment for smoking status, physical activity levels, ALT, AST, hypertension, diabetes, and SII. The restricted cubic splines (RCS) model was employed to assess the exposure‐response relationship between the DII, FII and the risk of MASLD. A logistic regression model was employed to examine the association between standardized insulin resistance markers and the risk of MASLD. Linear models were used to evaluate the relationships of the FII and the DII with insulin resistance markers. Prior to conducting causal mediation analysis, several key assumptions were established to ensure validity: the existence of causal relationships among the exposure variables (FII, DII), the mediator (insulin resistance markers), and the outcome (risk of MASLD); linear associations between these variables; and the absence of unmeasured confounding factors that could simultaneously affect both the mediator and the outcome. Based on these assumptions, a Bayesian approach was applied to perform causal mediation analysis with 1000 iterations of simulation to estimate indirect effects and their corresponding 95% confidence intervals. In addition, subgroup analyses were conducted to explore potential effect modifications. All analyses were conducted using R statistical software (version 4.3.3).

## 3 Result

### 3.1 Baseline Characteristics

This study incorporated a total of 13,675 participants; 7519 (55.21%) were female, 6133 (69.16%) were non‐Hispanic white, and the average age of all the participants was 48.56 years old, among whom 2522 (28.45%) were diagnosed with MASLD. Table 1 summarizes the detailed baseline characteristics based on MASLD status. Significant differences in the demographic and baseline clinical characteristics were observed between participants with MASLD and those without MASLD. Compared with participants without MASLD, those with MASLD were older, more often male, and had lower educational levels, PIR, and physical activity levels. The prevalences of diabetes and hypertension were higher among participants with MASLD. Liver function and SII were significantly higher in patients with MASLD. Comparative analysis of dietary inflammatory indices revealed significantly higher proinflammatory dietary profiles in MASLD participants, with elevated FII (−6.19 ± 0.06 vs. −5.97 ± 0.08) scores compared to participants without MASLD (all *p* < 0.001).

**TABLE 1:** Baseline characteristics of all participants were stratified by MASLD.

Variable	Total *N* = 13,675	Participants without MASLD *N* = 9784	Participants with MASLD *N* = 3891	*p*
Age	48.56 (0.28)	46.62 (0.31)	53.72 (0.36)	< 0.0001
BMI	28.49 (0.09)	26.35 (0.07)	34.16 (0.17)	< 0.0001
PIR	3.03 (0.03)	3.06 (0.03)	2.93 (0.04)	0.002
ALT	23.41 (0.14)	20.90 (0.14)	30.08 (0.36)	< 0.0001
AST	23.83 (0.13)	22.94 (0.15)	26.19 (0.26)	< 0.0001
SII	544.78 (4.34)	533.59 (5.14)	574.55 (6.66)	< 0.0001
FII	−6.13 (0.05)	−6.19 (0.06)	−5.97 (0.08)	< 0.001
DII	1.58 (0.03)	1.55 (0.03)	1.68 (0.04)	< 0.001
Physical activity	3315.58 (78.21)	3353.22 (82.74)	3215.45 (131.10)	0.001
Sex				< 0.0001
Female	7519 (55.21)	5682 (59.37)	1837 (44.14)	
Male	6156 (44.79)	4102 (40.63)	2054 (55.86)	
Race				< 0.0001
Mexican American	2190 (7.29)	1237 (6.04)	953 (10.62)	
Non‐Hispanic Black	2993 (11.57)	2506 (13.47)	487 (6.51)	
Non‐Hispanic White	6133 (69.16)	4294 (68.00)	1839 (72.24)	
Other	2359 (11.98)	1747 (12.49)	612 (10.63)	
Educational level				< 0.0001
Some college or AA degree	7059 (60.38)	5357 (62.82)	1702 (53.89)	
High school	5075 (33.63)	3545 (32.17)	1530 (37.51)	
Less than high school	1541 (5.99)	882 (5.01)	659 (8.59)	
Smoking status				< 0.0001
Former	3310 (24.51)	2110 (22.39)	1200 (31.44)	
Never	7894 (59.28)	5739 (61.49)	2155 (56.80)	
Now	1973 (14.69)	1504 (16.12)	469 (11.76)	
Hypertension				< 0.0001
No	7952 (62.10)	6390 (69.99)	1562 (41.22)	
Yes	5720 (37.86)	3391 (30.01)	2329 (58.78)	
Diabetes				< 0.0001
No	10,700 (83.97)	8379 (92.04)	2321 (67.13)	
Yes	2594 (14.61)	1072 (7.96)	1522 (32.87)	

Abbreviations: ALT, alanine aminotransferase; AST, aspartate aminotransferase; BMI, body mass index; DII, dietary inflammatory index; FII, food inflammatory index; MASLD, metabolic dysfunction‐associated steatotic liver disease; PIR, poverty income ratio; SII, systemic immune‐inflammation index.

### 3.2 Association Between Proinflammatory Dietary Indices and MASLD Risk and RCS Analysis

Multivariable logistic regression analyses revealed consistent positive associations between both proinflammatory dietary indices (DII and FII) with MASLD prevalence across all adjusted models (Table 2). In the fully adjusted model, for DII, each 1‐unit increase in DII score was associated with a 7% higher risk of MASLD (OR = 1.07, 95% CI: 1.04–1.10, *p* < 0.001). When DII was categorized into tertiles, participants in T3 showed 23% increased odds (OR = 1.23, 95% CI: 1.07–1.41), compared to participants in T1. As for FII, each 1‐unit increment in FII score corresponded to a 4% elevated MASLD risk (OR = 1.04, 95% CI: 1.02–1.06, *p* < 0.001) (Table 2). In tertile‐based analysis, the participants in T3 also demonstrated significantly increased odds (OR = 1.26, 95% CI: 1.09–1.47), compared to participants in T1. To further investigate the potential nonlinear associations between dietary inflammatory indices and MASLD, RCS analyses were performed after adjusting for potential confounding factors. As shown in Figure S1, both FII and DII were positively associated with the risk of MASLD in a dose–response pattern. However, the tests for nonlinearity were not statistically significant for either FII (*p* for nonlinearity = 0.329) or DII (*p* for nonlinearity = 0.318).

**TABLE 2:** Association of proinflammatory diet pattern with the risks of MASLD.

	Partially adjusted		Fully adjusted	
	OR (95% CI)	*p*	OR (95% CI)	*p*
FII				
Continuous	1.06 (1.04, 1.08)	< 0.0001	1.04 (1.02, 1.06)	< 0.0001
Tripartite				
T1 (13.64, −6.66)	ref		ref	
T2 (−6.66, −3.95)	1.14 (0.99, 1.31)	0.06	1.06 (0.92, 1.21)	0.432
T3 (−3.95, 0.42)	1.42 (1.23, 1.63)	< 0.0001	1.26 (1.09, 1.47)	0.002
*p* for trend		< 0.0001		0.003
DII				
Continuous	1.09 (1.05, 1.12)	< 0.0001	1.07 (1.04, 1.10)	< 0.001
Tripartite				
T1 (−4.44, 1.04)	ref		ref	
T2 (1.04, 2.73)	1.15 (1.00, 1.32)	0.056	1.10 (0.96, 1.27)	0.179
T3 (2.73, 5.50)	1.33 (1.17, 1.51)	< 0.0001	1.23 (1.07, 1.41)	0.0053
*p* for trend		< 0.0001		0.005

*Note:* Partially adjusted age, sex, race, PIR; fully adjusted smoking status, physical activity levels, hypertension, diabetes, and SII (based on partially adjusted).

Abbreviations: BMI, body mass index; CI, confidence interval; DII, dietary inflammatory Index; FII, food inflammatory index; MASLD, metabolic dysfunction‐associated steatotic liver disease; OR, odd ratio; PIR, poverty income ratio; SII, systemic immune‐inflammation index.

### 3.3 Association of Proinflammatory Dietary Indices and Biomarkers of Insulin Resistance

Table 3 summarizes the associations of dietary inflammatory indices (FII and DII) with biomarkers of insulin resistance. In the fully adjusted model, dietary inflammatory indices (FII and DII) were significantly positively associated with all biomarkers. Specifically, FII was positively associated with: BMI (β = 0.012, 95% CI: 0.001, 0.023, *p* = 0.034), TyG (β = 0.38, 95% CI: 0.26, 0.50, *p* < 0.001), TyG‐WC (β = 0.0009, 95% CI: 0.0004, 0.0014, *p* < 0.001), TyG‐WHtR (β = 0.21, 95% CI: 0.13, 0.29, *p* < 0.001), TyG‐ABSI (β = 3.55, 95% CI: 2.40, 4.69, *p* < 0.001), TyG‐BMI (β = 0.0021, 95% CI: 0.0009, 0.0032, *p* < 0.001). The DII was positively associated with: BMI (β = 0.019, 95% CI: 0.012, 0.025, *p* < 0.001), TyG (β = 0.12, 95% CI: 0.04, 0.19, *p* = 0.002), TyG‐WC (β = 0.0008, 95% CI: 0.0005, 0.0010, *p* < 0.001), TyG‐WHtR (β = 0.17, 95% CI: 0.13, 0.21, *p* < 0.001), TyG‐ABSI (β = 1.49, 95% CI: 0.83, 2.14, *p* < 0.001), TyG‐BMI (β = 0.0019, 95% CI: 0.0013, 0.0026, *p* < 0.001).

**TABLE 3:** Association of proinflammatory diet pattern with insulin resistance biomarkers.

	Partially adjusted, β (95% CI)	*p*	Fully adjusted, β (95% CI)	*p*
FII				
BMI	0.023 (0.013, 0.034)	< 0.0001	0.012 (0.001, 0.023)	0.034
TyG	0.42 (0.32, 0.52)	< 0.0001	0.38 (0.26, 0.50)	< 0.0001
TyG‐WC	0.0013 (0.0009, 0.0017)	< 0.0001	0.0009 (0.0004, 0.0014)	< 0.0001
TyG‐WHTR	0.27 (0.20, 0.33)	< 0.0001	0.21 (0.13, 0.29)	< 0.0001
TyG‐ABSI	3.81 (2.85, 4.76)	< 0.0001	3.55 (2.40, 4.69)	< 0.0001
TyG‐BMI	0.0032 (0.0021, 0.0042)	< 0.0001	0.0021 (0.0009, 0.0032)	< 0.0001
DII				
BMI	0.019 (0.013, 0.025)	< 0.0001	0.019 (0.012, 0.025)	< 0.0001
TyG	0.15 (0.09, 0.22)	< 0.0001	0.12 (0.04, 0.19)	0.002
TyG‐WC	0.0008 (0.0006, 0.0011)	< 0.0001	0.0008 (0.0005, 0.0010)	< 0.0001
TyG‐WHTR	0.17 (0.13, 0.21)	< 0.0001	0.17 (0.13, 0.21)	< 0.0001
TyG‐ABSI	1.73 (1.13, 2.32)	< 0.0001	1.49 (0.83, 2.14)	< 0.0001
TyG‐BMI	0.002 (0.0014, 0.0027)	< 0.0001	0.0019 (0.0013, 0.0026)	< 0.0001

*Note:* Partially adjusted age, sex, race, PIR; fully adjusted smoking status, physical activity levels, hypertension, diabetes, and SII (based on partially adjusted).

Abbreviations: BMI, body mass index; CI, confidence interval; DII, dietary inflammatory index; FII, food inflammatory index; MASLD, metabolic dysfunction‐associated steatotic liver disease; OR, odd ratio; PIR, poverty income ratio; SII, systemic immune‐inflammation index; TyG, triglyceride‐glucose index; TyG‐ABSI, triglyceride‐glucose‐a body shape index; TyG‐BMI, triglyceride‐glucose‐body mass index; TyG‐WC, triglyceride‐glucose‐waist circumference index; TyG‐WHtR, triglyceride‐glucose‐waist‐to‐height ratio.

### 3.4 Association Between Biomarkers of Insulin Resistance and MASLD Risk

Table 4 presents the associations between insulin resistance biomarkers and the risk of MASLD. In the fully adjusted model, all indicators, when analyzed as continuous variables, showed significant associations with MASLD risk. Among these, each standard deviation increase in TyG‐WC demonstrated the strongest association with MASLD risk (OR = 12.17; 95% CI: 10.57–14.01), followed closely by TyG‐WHtR (OR = 12.04; 95% CI: 10.52–13.78). TyG‐BMI also showed a substantial increase in risk (OR = 8.61; 95% CI: 7.67–9.66). More modest but still significant effects were observed for BMI (OR = 2.85; 95% CI: 2.58–3.15), TyG‐ABSI (OR = 2.85; 95% CI: 2.58–3.15), and the baseline TyG index (OR = 2.51; 95% CI: 2.26–2.79).

**TABLE 4:** Associations of insulin resistance biomarkers with the risks of MASLD.

	Partially adjusted	*p*	Fully adjusted	*p*
	OR (95% CI)		OR (95% CI)	
BMI	3.49 (3.21, 3.80)	< 0.0001	2.85 (2.58, 3.15)	< 0.0001
TyG	3.21 (2.94, 3.51)	< 0.0001	2.51 (2.26, 2.79)	< 0.0001
TyG‐WC	13.10 (11.49, 14.94)	< 0.0001	12.17 (10.57, 14.01)	< 0.0001
TyG‐WHtR	13.36 (11.76, 15.17)	< 0.0001	12.04 (10.52, 13.78)	< 0.0001
TyG‐BMI	9.75 (8.76, 10.84)	< 0.0001	8.61 (7.67, 9.66)	< 0.0001
TyG‐ABSI	3.49 (3.21, 3.80)	< 0.0001	2.85 (2.58, 3.15)	< 0.0001

*Note:* Partially adjusted age, sex, race, PIR; fully adjusted smoking status, physical activity levels, hypertension, diabetes, and SII (based on partially adjusted).

Abbreviations: BMI, body mass index; CI, confidence interval; DII, dietary inflammatory index; FII, food inflammatory index; MASLD, metabolic dysfunction‐associated steatotic liver disease; OR, odd ratio; PIR, poverty income ratio; SII, systemic immune‐inflammation index; TyG, triglyceride‐glucose index; TyG‐ABSI, triglyceride‐glucose‐a body shape index; TyG‐BMI, triglyceride‐glucose‐body mass index; TyG‐WC, triglyceride‐glucose‐waist circumference index; TyG‐WHtR, triglyceride‐glucose‐waist‐to‐height ratio.

### 3.5 Mediation Effect Analysis

**FIGURE 2:**
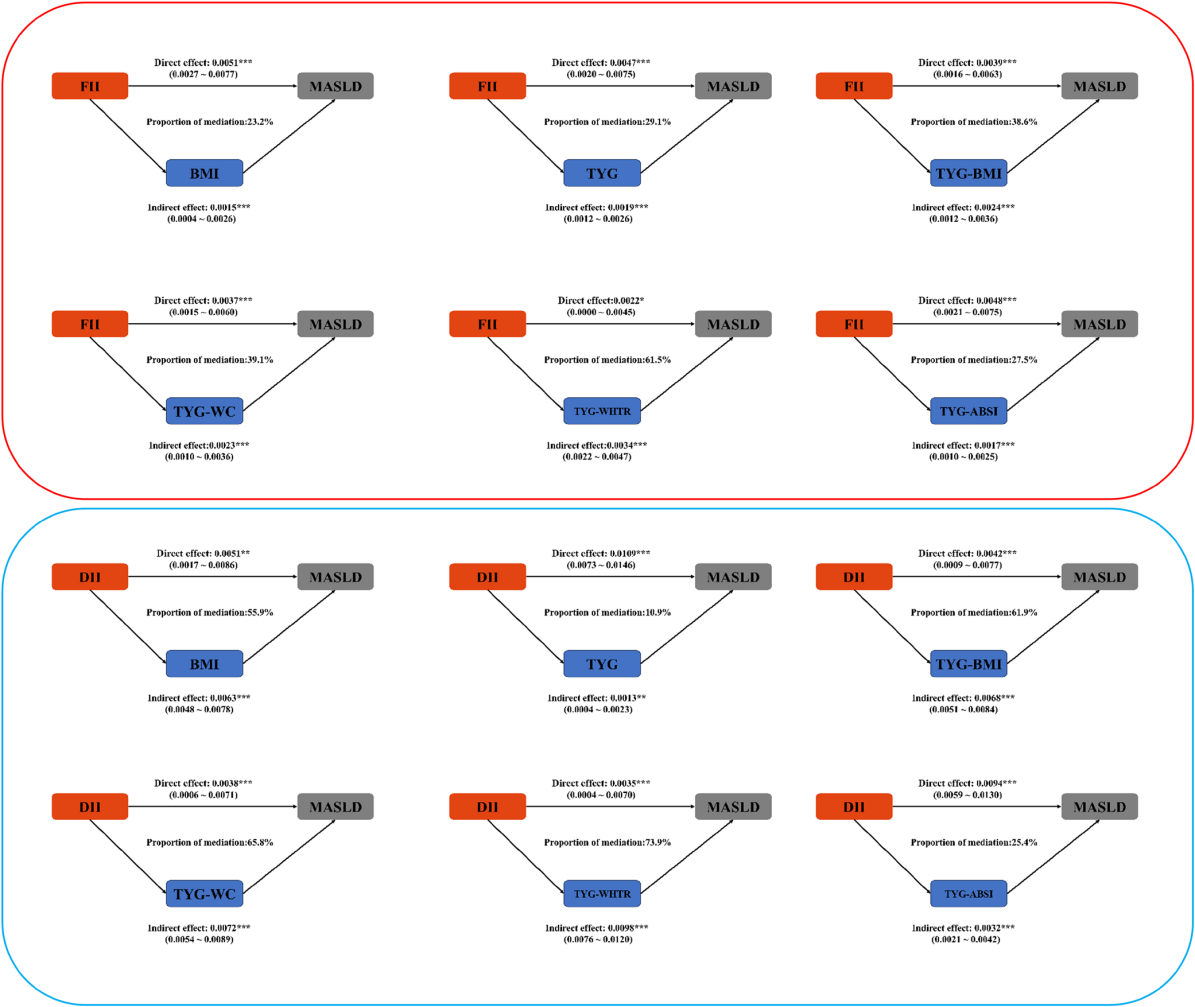
The mediation effects of IR biomarkers in the relationships of risks of MASLD. (A) Mediation analyses of insulin resistance biomarkers in the association between FII and the risk of MASLD. (B) Mediation analyses of insulin resistance biomarkers in the association between DII and the risk of MASLD.

Mediation analysis was conducted to assess the association of insulin resistance biomarkers in mediating FII and DII with the risk of MASLD (Figure 2). For FII, the proportion of the total effect mediated was highest for TyG‐WHtR (61.5%), followed by TyG‐BMI (39.1%), TyG‐WC (38.6%), TyG (29.1%), TyG‐ABSI (27.5%), and BMI (23.2%). A similar pattern was observed for DII, with TyG‐WHtR again exhibiting the largest mediating proportion at 73.9%. The remaining mediators showed proportions of 65.8% for TyG‐BMI, 61.9% for TyG‐WC, 55.9% for BMI, 25.4% for TyG‐ABSI, and 10.9% for TyG. Notably, TyG‐WHtR consistently accounted for the greatest mediated effect across both dietary indices.

### 3.6 Subgroup Analysis

**FIGURE 3:**
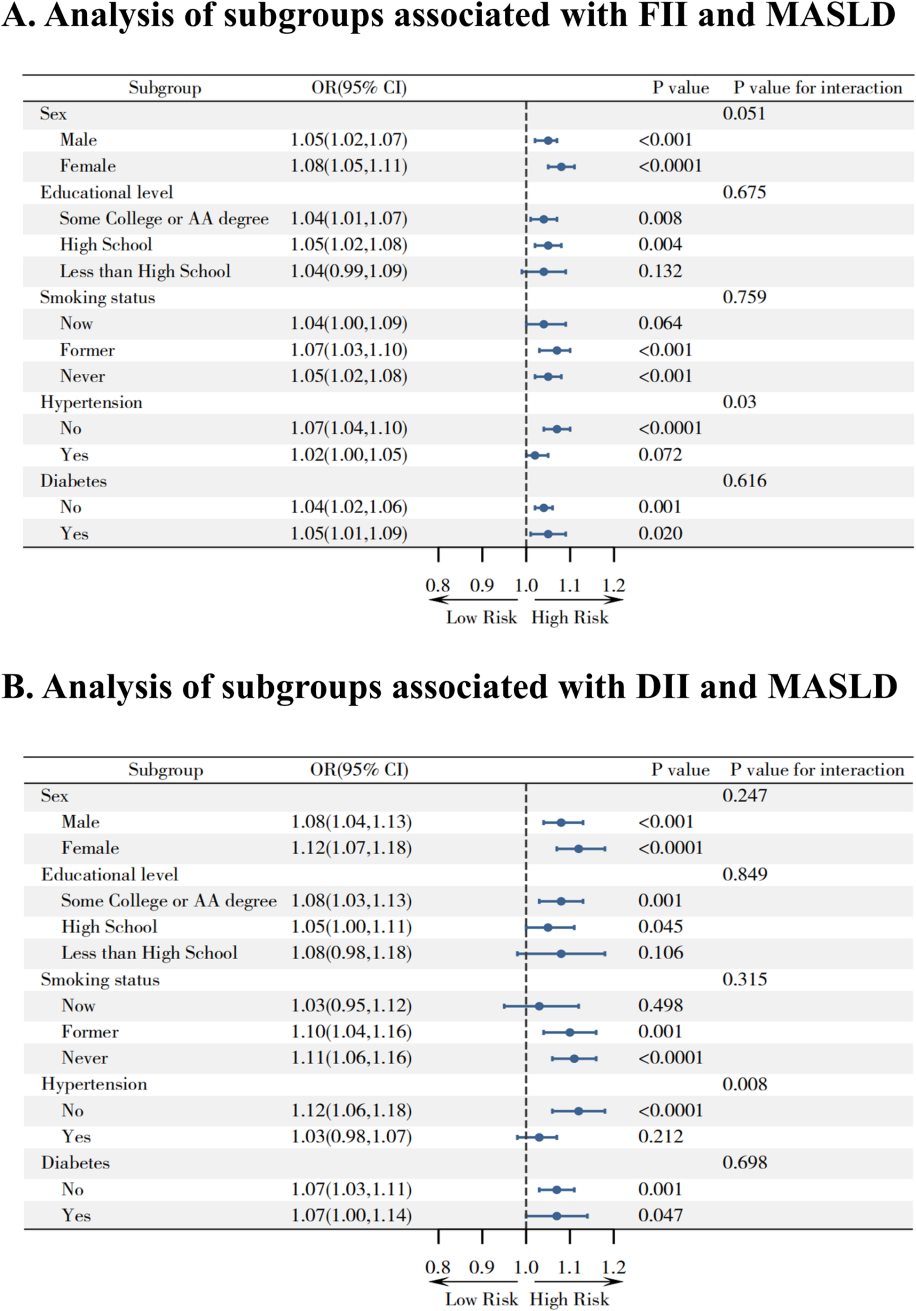
Subgroup analysis and interaction analysis (A) Subgroup analyses evaluating the association between FII and MASLD risk (B) Subgroup analyses evaluating the association between DII and MASLD risk.

As illustrated in Figure 3, the associations between both dietary indices (FII and DII) and the risk of MASLD were generally consistent with the overall effect across all predefined subgroups. Notably, however, these associations were markedly attenuated among current smokers and participants with hypertension. Furthermore, a statistically significant interaction effect was observed for hypertension.

## 4 Discussion

Based on a large, nationally representative sample of US adults, this study thoroughly investigates the complex relationships among proinflammatory diets, insulin resistance, and the risk of MASLD. Our key findings can be summarized as follows: (1) Proinflammatory dietary patterns (indicated by higher DII and FII scores) were independently associated with a higher prevalence of MASLD, exhibiting a dose–response relationship; (2) Proinflammatory diets were significantly correlated with the worsening of a range of insulin resistance biomarkers, including TyG and its composite indices; (3) Insulin resistance, particularly as characterized by TyG‐WHtR emerged as a key mediating pathway through which proinflammatory diets contribute to MASLD, accounting for over 60% of the association between the two.

Mounting evidence underscores the critical role of dietary patterns in the development and progression of MASLD (Hassani Zadeh et al. 2021). Proinflammatory diets, characterized by high intake of saturated fats, refined carbohydrates, and processed foods (Kim et al. 2022; Orliacq et al. 2023; Xu et al. 2024), are known to increase the risk of MASLD by elevating circulating levels of proinflammatory cytokines (e.g., TNF‐α, IL‐6) and reactive oxygen species (Miglio et al. 2013; Du et al. 2022). Conversely, diets rich in anti‐inflammatory components, including dietary fiber, polyphenols, and antioxidants, may help regulate immune responses and improve metabolic homeostasis. Our research confirms and extends the evidence regarding the relationship between dietary inflammatory potential and MASLD by demonstrating that proinflammatory dietary patterns, as measured by both DII and FII, are significantly associated with an elevated risk of MASLD. However, the effect size of FII is notably smaller than that of DII, a discrepancy that may be partially attributed to the inherent complexity of food interactions within dietary matrices. The DII may amplify single‐nutrient effects by ignoring food matrix interactions; FII's lower effect sizes likely reflect the real‐world complexity of dietary exposures. However, systemic inflammation represents only one potential pathway linking dietary inflammation to MASLD, and the limited explanatory contribution of individual inflammatory biomarkers suggests that additional metabolic pathways may play a substantial role.

The core finding of this study lies in revealing the key mediating role played by insulin resistance in this relationship. Mediation analysis indicated that insulin resistance explained 23.2%–73.9% of the association between proinflammatory diets and MASLD. Elevated BMI and insulin resistance have been well‐established as central pathophysiological drivers in the development and progression of MASLD (Luo and Lin 2021). The proinflammatory diets may trigger a systemic low‐grade inflammatory state, interfere with insulin signaling pathways (such as inducing IRS‐1 serine phosphorylation) (Hotamisligil 2006), and consequently induce insulin resistance in both the liver and peripheral tissues. Insulin resistance, in turn, leads to increased de novo lipogenesis in the liver, elevated influx of free fatty acids, and impaired β‐oxidation, ultimately promoting hepatocyte steatosis and the development of MASLD (Xu et al. 2022). It is particularly noteworthy that among all insulin resistance markers, the TyG‐WHtR demonstrated the strongest mediating effect (61.5% for FII and 73.9% for DII). Previous studies have shown that DII is significantly positively correlated with the risk of hyperlipidemia, and a higher DII score is also closely related to the deterioration of adult glucose homeostasis indicators (Farhangi et al. 2020; Han et al. 2023). Central obesity, as a clinical manifestation of visceral fat accumulation, can have its adipose tissue secrete a large amount of proinflammatory cytokines (such as TNF‐α, IL‐6) (Tilg et al. 2025), thereby exacerbating insulin resistance and metabolic inflammation. Therefore, the TyG‐WHtR essentially captures two core pathological processes: “metabolic disorders” and “central obesity” which likely explains its superior performance as a mediator. This finding provides important clinical implications: in assessing MASLD risk, composite indices that incorporate both metabolic parameters (blood lipids, glucose) and anthropometric measures (waist circumference, height)—such as the TyG‐WHtR—offer greater value than single indicators alone. Subgroup analysis revealed that the association between proinflammatory diets and MASLD was attenuated among smokers and patients with hypertension. This may be attributed to the fact that both smoking and hypertension are potent proinflammatory and proatherogenic factors (Hussain and Tripathi 2018; Mao et al. 2012; Mowry and Biancardi 2019), whose substantial effects on MASLD risk may “mask” the relative contribution of dietary inflammation. Furthermore, the significant interaction effect observed with hypertension suggests that for hypertensive individuals, controlling blood pressure itself may be more urgent and effective in reducing MASLD risk than dietary modifications alone, although maintaining a healthy diet remains crucial.

Our research has certain advantages. First, based on NHANES’ strict data collection procedures, complex probability sampling designs, and rigorous reviews, our research is representative. Based on this, we established for the first time a significant association between FII and MASLD risk and conducted mediation analysis, revealing the effects of biomarkers of insulin resistance in the proinflammatory dietary patterns (indicated by higher DII and FII scores)—MASLD association. However, this analysis has limitations inherent to cross‐sectional designs, including inability to establish temporal causality. While we adjusted for major confounders, residual confounding from unmeasured factors (e.g., genetic predisposition, specific nutrient intake) cannot be excluded. The use of US‐FLI rather than histology for MASLD diagnosis may underestimate cases with early‐stage steatosis.

## 5 Conclusion

Our study demonstrates that a proinflammatory diet is significantly associated with an increased risk of MASLD, and this association is primarily mediated by insulin resistance. These findings underscore the critical role of diet‐induced insulin resistance in the pathogenesis of MASLD, suggesting that dietary components driving systemic inflammation and metabolic dysfunction significantly contribute to disease development.

## Author Contributions

**Zongbiao Tan:** conceptualization, methodology, software, writing – original draft, investigation, visualization. **Yang Meng:** conceptualization, methodology, software, writing – original draft, visualization. **Haodong He:** conceptualization, methodology, software, writing – original draft, investigation, visualization. **Weiguo Dong:** writing – review and editing, funding acquisition, supervision, resources, project administration. **Jixiang Zhang:** methodology, validation, resources, data curation. **Yu pu:** methodology, validation, writing – review and editing, data curation. **Yanrui Wu:** methodology, validation, writing – review and editing, data curation.

## Funding

The authors have nothing to report.

## Ethics Statement

The NHANES study protocol was approved by the Institutional Review Board of the National Center for Health Statistics, with informed consent obtained from all participants. As our investigation is based exclusively on de‐identified, publicly released NHANES data, it qualifies for exemption from further IRB review under applicable regulatory guidelines.

## Consent

The authors have nothing to report.

## Conflicts of Interest

The authors declare no conflicts of interest.

## Supporting information


**Table S1:** The calculation method of derivative indicators representing insulin resistance.
**Figure S1:** RCS analyzed the relationship between proinflammatory dietary indicators and the risk of MASLD.

## Data Availability

The data employed in this analysis are derived from publicly accessible repositories and can be obtained via website https://www.cdc.gov/nchs/nhanes.
