# Supplementary material for: The Role of Insulin Resistance in Mediating the Association Between Proinflammatory Diets and Metabolic Dysfunction‐Associated Steatotic Liver Disease
Source: Food Sci Nutr. 2026 Aug 12;14(8):e72203. doi: 10.1002/fsn3.72203 (PMC13463420; doi:10.1002/fsn3.72203)
Supplement: Supplementary file 1 [file FSN3-14-e72203-s001.docx]

**The Role of Insulin Resistance in Mediating the Association Between Pro-Inflammatory Diets and Metabolic Dysfunction-Associated Steatotic Liver Disease**

Zongbiao Tan^1,#^, Haodong He^1,#^, Yang Meng^2,#^, Yu pu^3^, Yanrui Wu^1^, Jixiang Zhang^1^, Weiguo Dong ^1,✉^

**Affiliations**

1	Department of Gastroenterology, Renmin Hospital of Wuhan University, 238 Jiefang Road, Wuhan, China

2	State Key Laboratory of Ophthalmology, Zhongshan Ophthalmic Center, Sun Yat-sen University, Guangdong Provincial Key Laboratory of Ophthalmology and Visual Science, Guangzhou 510060, China

3	Department of General Practice, Renmin Hospital of Wuhan University, 238 Jiefang Road, Wuhan, China

^#^ These authors contributed equally to this work and shared first authorship.

^✉^ **Corresponding author**: Prof. Weiguo Dong, MD, Ph.D., Department of Gastroenterology, Renmin Hospital of Wuhan University, No 99 Zhangzhidong Road, Wuhan, 430060, Hubei Province, China. Email address: dongweiguo@whu.edu.cn, Tel: 027-88041911.

**Stable 1**. The calculation method of derivative indicators representing insulin resistance.

| Acronym | Full Name | Formula |
| --- | --- | --- |
| BMI | Body Mass Index | Weight(kg)/(Height (m))² |
| TyG | Triglyceride-Glucose Index | Ln [TG (mg/dL)×FG (mg/dL)/2] |
| TyG-WC | Triglyceride-Glucose-Waist Circumference Index | TyG Index×WC (cm) |
| TyG-BMI | Triglyceride-Glucose-Body Mass Index | TyG Index×BMI |
| TyG-ABSI | Triglyceride-Glucose-A Body Shape Index | TyG Index×[WC(m)/ (WC/ (BMI ^ ^(2/3)^ × Height ^ ^(1/2)^)] |
| TyG-WHtR | Triglyceride-Glucose-Waist-to-Height Ratio | TyG Index×(WC(cm) /Height(cm)) |

Abbreviations: TG: Fasting Triglycerides; FG: Fasting Glucose; WC: Waist Circumference; Ln: Natural Logarithm

**S Fiure 1.** RCS analyzed the relationship between pro-inflammatory dietary indicators and the risk of MASLD
